# Improving mental health and social participation outcomes in older adults with depression and anxiety: Study protocol for a randomised controlled trial

**DOI:** 10.1371/journal.pone.0269981

**Published:** 2022-06-27

**Authors:** Jessamine Tsan-Hsiang Chen, Viviana M. Wuthrich, Ronald M. Rapee, Brian Draper, Henry Brodaty, Henry Cutler, Lee-Fay Low, Andrew Georgiou, Carly Johnco, Michael Jones, Denise Meuldijk, Andrew Partington

**Affiliations:** 1 Centre for Emotional Health, School of Psychological Sciences, Faculty of Medicine, Health and Human Sciences, Macquarie University, Sydney, New South Wales, Australia; 2 Centre for Ageing, Cognition and Wellbeing, Faculty of Medicine, Health and Human Sciences, Macquarie University, Sydney, New South Wales, Australia; 3 Older Persons’ Mental Health Services, Prince of Wales Hospital, Sydney, New South Wales, Australia; 4 Centre of Healthy Brain Ageing, School of Psychiatry, Faculty of Medicine, University of New South Wales, Sydney, New South Wales, Australia; 5 Macquarie Centre for the Health Economy, Macquarie University, Sydney, New South Wales, Australia; 6 Ageing, Work and Health Research Unit, Faculty of Health Sciences, The University of Sydney, Sydney, New South Wales, Australia; 7 Centre for Health Systems and Safety Research, Australian Institute of Health Innovation, Macquarie University, Sydney, New South Wales, Australia; 8 Black Dog Institute, Faculty of Medicine, University of New South Wales, Sydney, New South Wales, Australia; University of Aveiro Department of Education and Psychology: Universidade de Aveiro Departamento de Educacao e Psicologia, PORTUGAL

## Abstract

**Background:**

Increasing both the frequency and quality of social interactions within treatments for anxiety and depressive disorders in older adults may improve their mental health outcomes and quality of life. This study aims to evaluate the clinical efficacy and cost utility of an enhanced cognitive behavioural therapy (CBT) plus social participation program in a sample of older adults with depression and/or anxiety.

**Methods:**

A total of 172 community-dwelling adults aged 65 years or older with an anxiety and/or depressive disorder will be randomly allocated to either an enhanced CBT plus social participation program (n = 86) or standard CBT (n = 86). Both treatments will be delivered during 12 weekly individual sessions utilising structured manuals and workbooks. Participants will be assessed at pre-treatment, post-treatment, and 12-month follow-up. The primary outcome evaluates mean change in clinician-rated diagnostic severity of anxiety and depressive disorders from baseline to post-treatment (primary endpoint) based on a semi-structured diagnostic interview. Secondary outcomes evaluate changes in symptomatology on self-report anxiety and depression measures, as well as changes in social/community participation, social network, and perceived social support, loneliness, quality of life, and use of health services. Economic benefits will be evaluated using a cost-utility analysis to derive the incremental cost utility ratios for the enhanced CBT program.

**Discussion:**

Outcomes from this study will provide support for the establishment of improved psychosocial treatment for older adults with anxiety and/or depression. Study outcomes will also provide health systems with a clear means to reduce the impact of poor emotional health in older age and its associated economic burden. In addition to the empirical validation of a novel treatment, the current study will contribute to the current understanding of the role of social participation in older adult wellbeing.

**Trial registration:**

Prospectively registered on the Australian New Zealand Clinical Trials Registry (ID: ACTRN12619000242123; registered 19^th^ February 2019) and the ISRCTN registry (ID: ISRCTN78951376; registered 10^th^ July 2019).

## 1. Introduction

### 1.1 Background and rationale

Untreated depression and anxiety in older populations are associated with poor physical health, disability, morbidity, increased costs of service use and medications, as well as greater risk of cognitive decline [1–4]. Furthermore, depressive and anxiety disorders are associated with reduced social participation, poor social support, as well as increased feelings of loneliness and isolation [5–7], which in turn exacerbate risk for suicidal ideation, self-harm, suicide, and death [8–10]. Consequently, the economic costs of these disorders for healthcare systems and society are high [11], especially considering the rapidly ageing population [12].

Anxiety and depression can be successfully treated in older adults using psychological interventions, with the strongest evidence for cognitive behavioural therapy (CBT) [13–15]. In a randomised controlled trial (RCT), adults aged 60 years and older with comorbid anxiety and depression who completed a transdiagnostic CBT program showed large reductions in diagnostic severity for anxiety disorders and unipolar depression compared to a waitlist control condition [16]. In another RCT, this transdiagnostic CBT program also led to faster and sustained improvements in anxiety and depression on diagnostic severity in older adults compared to a discussion group. Furthermore, more than half of participants remitted from their main presenting diagnosis, and almost 40% were remitted from all depressive and anxiety disorders at six months post-treatment [17]. Taken together, these results demonstrate the efficacy of transdiagnostic CBT in reducing anxiety and depression in older adults. However, despite these promising results, up to half of older adults continue to experience clinically impairing symptoms of depression and anxiety following CBT [17].

Poor social connectedness and reduced social participation might be related to suboptimal treatment outcomes. Social participation broadly refers to the degree to which a person participates in society through friendships, social groups, and volunteering [5]. Older adults with anxiety and depression have reduced social participation, poorer levels of perceived social support, and increased feelings of loneliness and isolation [5–7]. Importantly, the relationship between social interactions and emotional distress is likely bidirectional [6]. That is, heightened emotional distress may exacerbate cardinal symptoms of anxiety and depression, such as irritability, avoidance of social interactions, social withdrawal, anhedonia, and negative cognitive biases, which can lead to reduced or unsatisfying social interactions [18]. In turn, heightened sensitivity to social rejection as a result of poor social functioning could also maintain symptoms of anxiety and depression [5, 19, 20].

Despite the evidence and theory linking reduced social participation with the maintenance of anxiety and depression, few studies have examined the impact of directly targeting social factors (social network, relationship satisfaction) on mental health outcomes in older adults. A short-term behavioural activation delivered using videoconferencing has been found to be associated with greater improvements in social connectedness at 12-week follow-up compared to a control group which delivered supportive visiting (without direct coaching of specific coping skill development) also delivered via videoconferencing [21]. Another intervention that focused on addressing psychosocial barriers, including low social self-efficacy and environmental barriers, has shown potential benefits in reducing loneliness in older adults compared with a no-treatment control group [19]. To date, no studies have directly evaluated the effect of targeting the broader concept of social participation, including participation through friendships, social groups, and volunteering, or the impact of targeting this alongside traditional treatment approaches for mental ill-health (e.g., CBT). It is possible that standard treatments for anxiety and depression in older adults would benefit from a more direct and targeted focus on increasing social participation compared to standard CBT intervention.

## 2. Materials and methods

### 2.1 Objectives

The main aim of this study is to evaluate the clinical efficacy and cost utility of an augmented psychological CBT intervention for treating anxiety and/or depression in older adults. We will compare this augmented psychological intervention, which will incorporate a targeted focus on increasing social participation (enhanced CBT), with the current best practice transdiagnostic CBT program (standard CBT) for older adults with anxiety and/or depression [16, 17]. To evaluate the efficacy of the enhanced CBT program, the inclusion criteria for study participants will be selective; participant adherence will be closely monitored through clinician supervision, and the clinicians delivering treatment will be specially trained in the intervention and its application. The intervention will be conducted in our research laboratory and the risk of other confounding interventions (e.g., concurrent treatments) will be limited. The enhanced CBT program has the potential to increase recovery rates for older adults suffering from depressive and anxiety disorders, enhance wellbeing and social participation in this age group, and improve economic outcomes for payers and society. The results of this study could improve the efficacy of psychological interventions for late-life anxiety and depression, in a way that offers value-for-money and could be considered for public financing. In addition, we will examine the underlying moderators of treatment response, and changes in cognitive functioning over time.

### 2.2 Trial design

**Fig 1:**
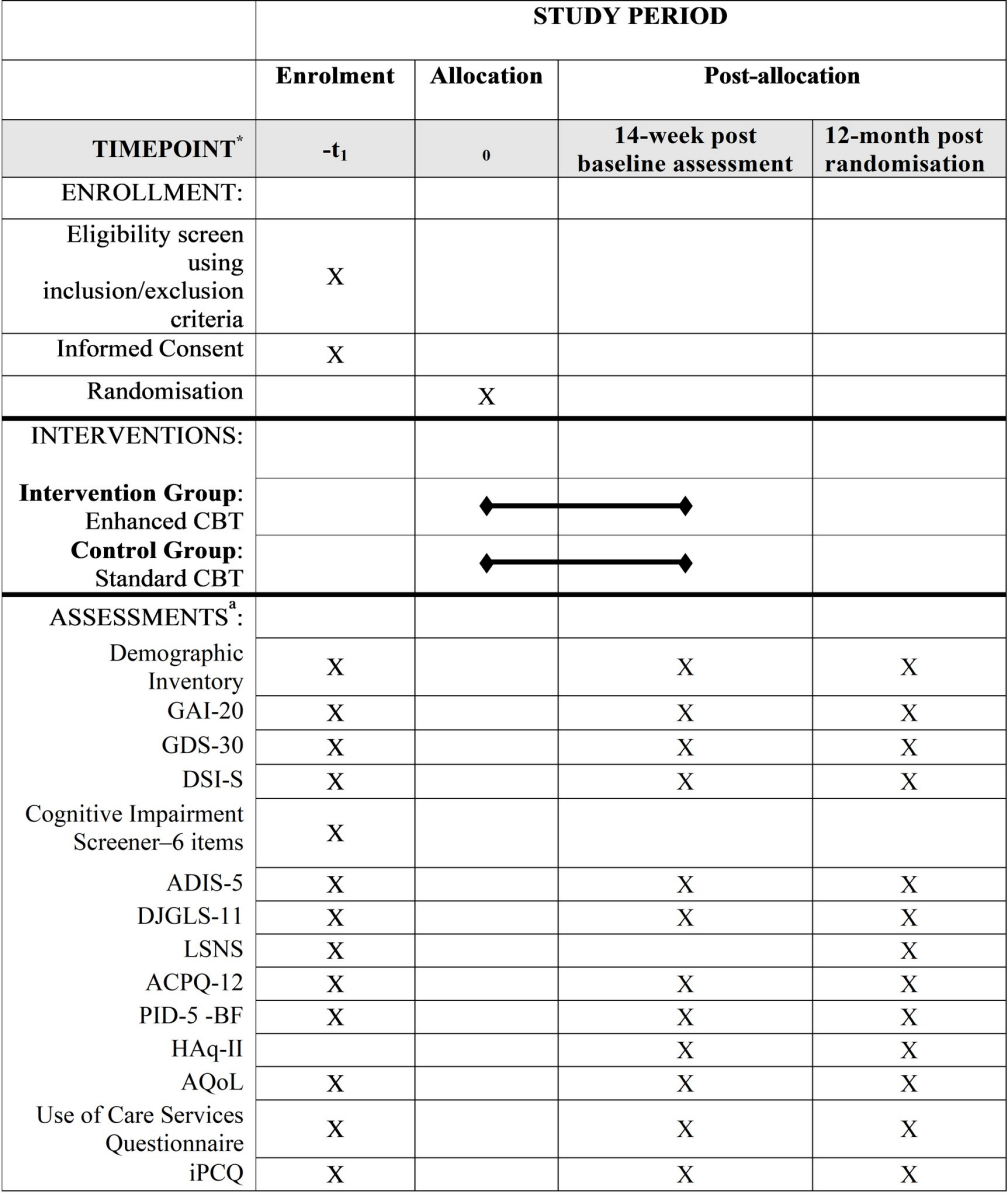
SPIRIT schedule of enrolment, interventions, and assessments. *-t1 = Baseline assessment. NOTE: GAI-20 = Geriatric Anxiety Inventory-20 items; GDS-30 = Geriatric Depression Scale-30 items; DSI-S = Depressive Symptoms Inventory–Suicide Subscale; ADIS-5 = The Anxiety Disorders Interview Schedule 5th edition; DJGLS-1 1 = De Jong Gierveld Loneliness Scales-11 items; LSNS = Lubben Social Network Scale; ACPQ-12 = Australian Community Participation Questionnaire-12 items; PID-5–BF = The Personality Inventory for DSM-5 Brief Form; HAq-II = Helping Alliance Questionnaire; AQoL = Assessment of Quality of Life; iPCQ = iMTA Productivity Cost Questionnaire. ^a^Measures will be administered over the phone, online or paper based (participant’s choice).

This study uses a parallel-group superiority randomised controlled trial design to evaluate the clinical efficacy and cost utility of an enhanced CBT program that will comply with the procedures outlined in the CONSORT statement and its extensions, and the Standard Protocol Items: Recommendations for Interventional Trials [SPIRIT; 22]. Eligible participants who provide informed consent are randomly assigned to one of two groups using 1:1 allocation: the control group (Standard CBT) or the intervention group (Enhanced CBT). See Fig 1 for a SPIRIT schedule of enrolment, interventions, and assessments.

### 2.3 Study setting

The trial will run through the Macquarie University Centre for Emotional Health Clinic (CEHC), Sydney, Australia. The CEHC has built a strong reputation for psychological treatments and a wide referral base over the past 15 years. In particular, the CEHC has developed a growing reputation for the treatment of older adult anxiety and depression over the past seven years and has a regular referral flow of patients.

### 2.4 Eligibility criteria

Eligible participants are adults aged 65 years or older with a primary diagnosis of an anxiety and/or unipolar depressive disorder according to the Diagnostic and Statistical Manual of Mental Disorders– 5^th^ Edition (DSM-5) [23] criteria assessed by the Anxiety Disorders Interview Schedule for DSM-5 (ADIS-5) [24]. In addition, participants will need to have adequate understanding of the English language as indicated by the ability to read a local newspaper.

Individuals will be excluded if they have current psychotic or bipolar traits, active suicidal intent, significant uncorrected hearing loss and likely moderate to severe dementia as screened by the six-item Cognitive Impairment Screener [25]. Participants currently using psychotropic medications (e.g., anxiolytic and antidepressant medication) will not be excluded, but participants will need to be stabilised on medications for one month before baseline assessment. Participants will report medication type, dosage and frequency of use, and whether the medications were prescribed for management of depression or anxiety. All medication changes will be noted at each assessment and those related to anxiety and mood management will be statistically controlled in analyses.

### 2.5 Interventions

Both the standard and enhanced CBT programs are guided by structured manuals for therapists and supported by workbooks containing homework exercises and lesson summaries for participants. In both treatment conditions, sessions will be run by clinical psychologists and provisional psychologists trained in delivery of the treatment protocols. To control for therapist differences, all therapists will be trained in and will conduct both treatments (allocated randomly). Supervision will be provided by chief investigators (CIs) and associate investigators (AIs) with treatment adherence and differentiation between conditions as a core focus. All therapy sessions will be recorded, and a random 20% will be rated by an independent expert unaware of the study hypotheses for adherence to the therapeutic manual using a codebook and form based on Waltz, Addis, Koerner, & Jacobson [26]. Therapy alliance will be measured using the 19-item Helping Alliance Questionnaire [HAq-II; 27].

The standard program will comprise our empirically validated CBT program for older age anxiety and depression, *Ageing Wisely*. The *Ageing Wisely* program consists of 12 weekly individual one-hour sessions to teach practical skills to help manage anxiety and depression including: goal setting, activity scheduling, problem solving, graded exposure, cognitive restructuring, and sleep hygiene. Skills such as assertive communication training, as well as examples that illustrate application of CBT skills to increase social connections, were not included in the standard CBT program. Homework activities are assigned to promote skill acquisition and generalisation.

The enhanced CBT program also comprises 12 weekly individual one-hour sessions, teaching the same CBT skills as the standard *Ageing Wisely* program in addition to assertiveness training, however all skills include a strong focus and application to bolstering social participation and connections. Specifically, the importance of bolstering social connections is highlighted in treatment rationale. Participants will be encouraged to 1) include regular social activities in activity scheduling; 2) apply cognitive restructuring to shift unrealistic cognitions relating to social situations and interpersonal relationships; 3) reduce avoidance of social situations or being unassertive through the practice of graded exposure; 4) apply problem-solving to overcome barriers to participating in social activities; and 5) practise assertive communication skills to reduce interpersonal conflict. It should be noted that the addition of assertiveness training and other social intervention does not reduce the overall provision of CBT to participants in the enhanced CBT condition. Rather, these skills will be taught in the context of CBT, in that participants are encouraged to identify and challenge maladaptive cognition specifically with respect to social interaction, reduce avoidance of social participation or being non-assertive, and problem-solve any barriers to practising assertive communication. Further explicit skills are taught to increase formation of new social relationships and participate in new social group activities.

### 2.6 Outcomes and participant timeline

The study has a longitudinal design conducted over 12 months with three assessment points. Outcome measures will be collected at baseline (T1) (prior to randomisation); post-treatment (T2) (14 weeks from baseline assessment); and 12-month follow-up (T3) (12 months from start of therapy) by blinded assessors. The primary and secondary outcome measures are presented in Table 1. Measures include self-reported measures and measures administered by an independent assessor (independent clinician) blind to treatment allocation at each of the time points. In addition to blinding of treatment type by the research assistant, at the start of each assessment participants will be asked not to confer the details of their treatment, including treatment condition, to the independent assessors. Clinician-report measures (treating clinician) are also administered.

**Table 1:** Primary and secondary outcome measures by time interval.

Instrument	Domain	Type^a^	Time Interval^b^^a2^			Reference
			T_1_	T_2_	T_3_	
Demographic Inventory	Demographics	SR	✓	✓	✓	
GAI-20	Anxiety	SR	✓	✓	✓	[28]
GDS-30	Depression	SR	✓	✓	✓	[29]
DSI-S	Depression/Suicide	SR	✓	✓	✓	[30]
ADIS-5	Psychopathology	BA	✓	✓	✓	[24]
DJGLS-11	Social Connection	SR	✓	✓	✓	[31]
LSNS	Social Network	SR	✓	✓	✓	[32]
ACPQ-12	Social Participation	SR	✓	✓	✓	[33]
PID-5 -BF	Personality Traits	SR	✓	✓	✓	[34]
HAq-II	Therapeutic Alliance	SR		✓		[27]
AQoL	Health Related Quality of Life	SR	✓	✓	✓	[35]
Use of Care Services Questionnaire	Cost-Effectiveness	SR	✓	✓	✓	N/A
iPCQ	Cost-Effectiveness	SR	✓	✓	✓	[36]

NOTE: GAI-20 = Geriatric Anxiety Inventory– 20 items; GDS-30 = Geriatric Depression Scale– 30 items; DSI-S = Depressive Symptoms Inventory–Suicide Subscale; ADIS-5 = The Anxiety Disorders Interview Schedule 5^th^ edition; DJGLS-11 = De Jong Gierveld Loneliness Scales—11 items; Lubben Social Network Scale; ACPQ-12 = Australian Community Participation Questionnaire– 12 item; PID-5 –BF = The Personality Inventory for DSM– 5; HAq-II = Helping Alliance Questionnaire; AQoL = Assessment of Quality of Life; iPCQ = iMTA Productivity Cost Questionnaire.

^a^SR = Self-eeport scales administered by participants; OS = Observer scale administered by treating clinician; BA = Scale administered by independent blind assessor

^b^T_1_ = Baseline assessment; T_2_ = 14-week post-treatment assessment; T_3_ = 12-month follow-up assessment.

The primary outcome is the mean changes in Clinical Severity Rating (CSR) across all anxiety and depressive disorders on the ADIS-5 [24] at post-treatment. The ADIS -5 is a semi-structured interview for diagnosing psychiatric conditions according to DSM -5 [23] criteria. The CSR provides an overall severity rating comprising the level of distress and impairment associated with each DSM disorder from 0 (none) to 8 (very severe). A CSR of 4 or higher indicates that the disorder meets or exceeds the threshold for a formal DSM-5 diagnosis. The mean severity rating is calculated to examine overall changes in diagnostic severity over time. This interview will be administered by blinded clinical psychologists, psychologists, or provisional psychologists who will be trained in the interview administration and receive regular supervision to assist with diagnostic decisions.

Secondary outcomes will be measured by:

1) Mean changes in CSR across all anxiety and depressive disorders based on the ADIS-5 at 12-month follow-up.
2) Changes in symptomatology at post-treatment and 12-month follow-up on the following measures:
  **Geriatric Anxiety Inventory** [GAI; 28]. The GAI is a 20-item self-report measure of anxiety in older adults.
  **Geriatric Depression Scale** [GDS; 29]. The GDS is a 30-item self-report measure of depression for use in older adult populations.
  **Depressive Symptoms Inventory–Suicide Subscale** [30] is a 4-item measure of suicidal ideation.
2) Changes in social participation/connection at post-treatment and 12-month follow-up on the following measures:
  **De Jong Gierveld Loneliness Scales** [31], which measures feelings of loneliness and
  perceived social isolation with 11 items.
  **Lubben Social Network Scale** [LSNS; 32]. The LSNS is a commonly used tool to assess social connections in older adult populations. The 18-item scale assesses the frequency and quality of family, friendship and neighbour support.
  **The Australian Community Participation Questionnaire-Short Form [33]** The 12-item short form of the ACPQ measures frequency of participation in seven different social activities (contact with extended family, friends and neighbours, contact with friends and family, attending organised community events, volunteering and political protest).
3) Cost utility of the interventions on the following measures:
  **Assessment of Quality of Life** [AQoL-8D; 35, 37]. The AQoL-8D measures quality of life and health outcomes across eight domains (independent living, relationships, mental health, coping, pain, senses, self-worth, and happiness).
  **Use of Care Services.** The Use of Service survey is developed by the research team to assess use of health and community care services in the preceding six months, including hospital services, medical services, allied health services, community-based services, and diagnostic services, supplemented with linked data from the NSW Centre for Health Record Linkage (CHeReL) on Admitted Patient data, Emergency Department data, and Mental Health Ambulatory data, along with Medicare Benefit Schedule (MBS) and Pharmaceutical Benefit Schedule (PBS) data.
  **iMTA Productivity Cost Questionnaire** [iPCQ; 36]. The iPCQ is a standardised instrument to measure and value health-related productivity losses including presenteeism and absenteeism in paid and unpaid work.

In addition to the primary and secondary outcome measures, the **Personality Inventory for DSM-5—Brief Form** [PID-5-BF; 34] will be used to undertake a mediation analysis on treatment outcomes. The PID-5-BF is a 25-item self- rated personality trait assessment scale for adults aged 18 years and older. It assesses five personality trait domains including negative affect, detachment, antagonism, disinhibition, and psychoticism, with each trait domain consisting of five items.

### 2.7 Sample size

Based on review of the available literature and our pilot study results comparing this enhanced CBT program to an active control group, we predict the enhanced CBT program to produce a controlled effect size (against standard intervention) of Cohen’s *d* = 0.5. Therefore, based on a moderate effect of Cohen’s *d* = 0.5, with power at 90% and, alpha at 0.05 (two-sided), 172 participants (86 equally randomised to each condition) in total are required, with the expectation that 15% of participants will be unsuitable based on outcomes of the baseline assessment (based on previous studies in our clinic that use a brief intake screener to determine likely eligibility into the program prior to baseline assessment), at least 200 participants will be recruited. In order to understand the representativeness of the population, demographic information will be collected with respect to sex, age, country of birth, ethnicity, education, marital status, income, employment status, living circumstances (e.g., retirement villages), pre-existing health conditions, psychiatric history (including duration), and current medications (type, dosage, and frequency) at time of intake/baseline assessment, post-treatment, and at each follow-up assessment.

### 2.8 Recruitment

Participants will be recruited to the CEHC from general practices, primary health network, other health professional or health organisations (e.g., aged care, hospital, residential aged care). Participants can self-refer. In addition, the study will be advertised broadly to the community and general practitioners (GPs), using brochures, media and newspaper stories and advertisements, and through Beyond Blue’s professional networks. The GPs in the local area will be informed about the study and provided with a brochure about how to improve detection and screening of anxiety and depression in their older patients.

Following referral, older adults will be screened over the telephone. Intake officers will assess likely suitability for the trial based on the inclusion and exclusion criteria outlined in Section 2.4. Older adults who are likely to meet the inclusion criteria will be sent participant information and consent forms. Intake officers will follow up to review understanding of the participant information and consent forms and to answer any questions. Older adults who are interested in participating will be asked to provide written informed consent, followed by completing full baseline measures according to the study design, including the ADIS administered by clinical psychologists, psychologists, or provisional psychologists at the Macquarie University CEHC. All participants will be allocated a deidentified alpha-numeric code by the research team. If a participant is deemed eligible after completion of the baseline assessment, they will be randomly allocated to one of the two treatment conditions by the research team (see Section 2.9 Assignment of intervention), and an appointment will be scheduled for the first treatment session. The average time between randomisation and entry into therapy is estimated to be two to four weeks depending on participant flow and clinician availability. If an older adult is ineligible for enrolment in the current study, the primary reason of ineligibility will be documented. Participants who do not consent or do not meet eligibility criteria will be offered alternative services at the CEHC as appropriate and/or referred to alternative services in the local area and/or to their GP.

### 2.9 Assignment of intervention

Eligible participants will be randomly allocated to receive enhanced CBT or standard CBT in a 1:1 ratio. The randomisation schedule will be generated using a computerised randomiser (www.randomizer.org) and placed into consecutively numbered sealed envelopes by an independent statistician. Block randomisation will be used to ensure that the two comparison groups are the same size.

### 2.10 Data collection methods

The clinician involved in the treatment of the participant will administer all the clinician-based instruments during treatment. Self-report measures are collected online, via pen-and-paper or over the telephone, at the participant’s choice. The semi-structured diagnostic interview (ADIS-5) will be administered at baseline, post-treatment, and at 12-month follow-up by independent assessors who are blind to treatment allocation. All participant data will be labelled using a deidentified alpha numeric code to ensure confidentiality.

All randomised participants will be followed up for data collection purposes at each time point three times using multiple formats (telephone messages, email and mail). If a participant misses an assessment time point, or if the intervention deviates from the protocol, participants will remain in the study and will be followed up for any subsequent assessments. Data collection will be discontinued for participants who formally withdraw their consent to participate, or who otherwise experience changes in their lives that preclude or complicate further participation; however, all data collected will remain in the study and be included in the analyses. Wherever possible, information about their decision to withdraw from the study will be collected to comply with CONSORT recommendations and facilitate correct reporting of adverse events.

### 2.11 Data management and confidentiality

To safeguard the confidentiality of the participants and to ensure proper handling of all data, processing of all data reporting complies with CONSORT recommendations and is in accordance with the National Statement on Ethical Conduct in Human Research [38]. Participants will provide electronic or written informed consent. All participant data will be allocated a deidentified code so that confidentiality is maintained during the study, and when the data are stored. Deidentified data will be stored in password protected secure online data storage repositories indefinitely at Macquarie University. Only chief investigators, the independent biostatistician, the project manager, and research assistant will have access to this deidentified data in order to facilitate assessment and welfare of participants in a crisis (telephone number, email address). This information is stored in a separate password protected file on the Macquarie University server. Linked resource utilisation data will be extracted by the NSW Centre for Health Record Linkage (CHeReL) and the Commonwealth Department of Human Services, using the personal identifiers of consenting study participants. The content data from the routine data collections will be returned to the research team in a deidentified format, so that it can be merged with trial data using the study specific IDs. This is in line with the ‘separation principle’ that ensures the maintenance of distinct and disjointed research datasets that mitigate re-identification risks.

### 2.12 Statistical methods

Data will be analysed using intention-to-treat principles. All participants will be included in analyses based on the condition to which they were initially allocated. Multiple imputation will be used to manage missing data. The efficacy of the programs will be established using mixed model analysis to compare the mean differences in clinical diagnostic severity (established by clinicians who are blind to treatment allocation) and scores on self-report measures at pre-treatment, post-treatment, and at a 12-month follow-up period.

Analysis will be a mixed model with random subject effect, fixed effects relating to group allocation (between-group), time (within-subject) and covariates. Interaction contrasts will be used if the time x group interaction reaches statistical significance, but the main effect contrast will be used if not.

Statistical inference will be based on the nonparametric bootstrap if the assumption of Normality is violated. The same process will be used for the secondary hypotheses to compare the groups on all outcome measures. Differences between groups in baseline characteristics, and therapy factors (e.g., sessions attended, therapist alliance) will be examined and controlled for in analyses.

In addition, an economic evaluation will be undertaken to measure the relative benefit in health outcomes and resource use for the enhanced intervention compared to standard CBT. Moderation analyses will examine the impact of moderators on treatment outcomes.

#### 2.12.1 Mediation analyses

Mediation will be evaluated using path models. Of key interest are the total effect (sum of all paths between clinical severity rating at baseline and 12-month follow-up), the direct path between clinical severity rating at baseline and 12-month follow-up, and the indirect path (total-direct) via key demographic variables (e.g., age, gender), personality and social participation factors. Statistical inference will be based on the nonparametric bootstrap if the assumption of normality is violated.

### 2.13 Economic evaluation

An economic evaluation will be conducted from the healthcare system perspective. Health outcomes captured through the AQoL-8D will be evaluated using Quality Adjusted Life Years (QALYs). Intervention and resulting costs per patient in the enhanced CBT group will be compared against those in the standard CBT group, and include acute care, primary care, mental health care, community care, and medication use. Data collected using the Use of Care Services survey will be supplemented with linked, individual-level Admitted Patient data, Emergency Department data and Mental Health Ambulatory data, along with Australian Government data from the Medicare Benefit Schedule (MBS) and Pharmaceutical Benefit Scheme (PBS). A cost-utility analysis will compare differences in QALYs with differences in resource use, and will be presented using an incremental cost utility ratio. This will provide a basis to make judgements on whether the enhanced CBT is cost-effective given to the price that Australian Government funders are likely willing to pay for a QALY for the target population [39]. The *iMTA Productivity Cost Questionnaire* [36] with the Australian Community Participation Questionnaire [33] will be used to estimate the value of social contributions.

### 2.14 Oversight and monitoring

#### 2.14.1 Data monitoring, harm, and auditing

Data management will be overseen by an independent data safety and monitoring board (DSMB) comprising a biostatistician, clinical trial expert, and health information systems expert. The DSMB committee will provide their expertise and recommendations to the research group. The primary responsibilities of the DSMB are to 1) periodically review and evaluate the accumulated study data for participant safety and progress, and, when appropriate, efficacy, and 2) make recommendations to the research group concerning the continuation, modification, or termination of the trial. In addition, a clinical advisory committee including a geriatric psychiatrist, clinical psychologist and consumer advocates will meet prior to, and six-monthly, for the trial duration. The CIs will report to the independent DSMB on all adverse events, as well as to the Ethics committee.

#### 2.14.2 Adverse events reporting

Any adverse events or clinical complications that arise as result of the therapeutic product offered will be reported by clinicians, managers, and research assistants to CIs during regular project supervision. All adverse events will be documented and reported in the trial outcomes, and the Independent Advisory and Data Safety Boards will be informed. All serious adverse events will be reported to the HRECs within 72 hours. The reports will be followed by a detailed written report. Follow-up reports will identify a participant by their unique code.

#### 2.14.3 Ethics and dissemination

This study has been approved by the Macquarie University Human Research Ethics Committee (Reference: 5201938336887; approved 24/01/2019), the NSW Population & Health Services Research Ethics Committee (PHSREC) (Reference: 2019/ETH12532), and the External Request Evaluation Committee (EREC) (Reference: RMS0283) within the Australian Department of Human Services. Changes to the trial will be submitted as amendments. See section ‘Data Management’ for information on consent, confidentiality, and access to data. The results of the study will be disseminated in peer-reviewed journals and presented at (inter)national conferences. Results will be reported on institutional websites and consumer-based websites. In all cases, only deidentified data will be used, and summary statistics will be reported. No information identifying individual participants or sites of service delivery, will be reported. Study records will be retained for a minimum of 15 years after the last publication arising from the study.

#### 2.14.4 Trial registration

This study has been registered on the Australian New Zealand Clinical Trials Registry (ANZCTR) (ID: ACTRN12619000242123; registered 19^th^ February 2019) and the International Standard Randomised Controlled Trial Number (ISRCTN) (ID: ISRCTN78951376; registered 10^th^ July 2019) prior to enrolment of the first participant.

## 3. Discussion

Emerging research indicates that increasing both the frequency and quality of social interactions may be key to maintaining healthy ageing [5]. Given the likely bidirectional relationships between social participation and emotional distress [6], interventions that enhance social participation through increased meaningful social contact, social group membership and volunteering should lead to increased quality of life, improved physical and mental health, as well as reduced mortality in older adults. By building stronger social support and increasing social contact, the maintenance of psychological intervention outcomes for older adults is also likely to be enhanced. This study seeks to evaluate the clinical efficacy and cost utility of incorporating a strong focus on social participation into a standard CBT program for older adults with clinical levels of anxiety and depression.

Participants in the enhanced CBT program will receive current best-practice transdiagnostic CBT, in addition to being actively encouraged to make new social connections and/or strengthen existing social connections. One major strength of our intervention design is that the enhanced CBT program will target maladaptive cognitions with respect to social interactions. In light of previous findings that up to half of older adults continue to experience clinically impairing symptoms of depression and anxiety following CBT [17], there are likely at least two groups of non-responders. The first is individuals who show an inadequate response at post-treatment, the second is those who initially respond to treatment but quickly relapse. In either case, poor social connectedness might be one factor related to suboptimal treatment outcomes. Loneliness research has posited that the perception of social isolation potentially increases an implicit hypervigilance for social threats, as well as biased appraisal of other people’s intentions and behaviours [40, 41]. These maladaptive biases are likely to influence an individual’s motivation to engage in social interactions [21], as well as triggering maladaptive behaviours in social situations, and social withdrawal. Further, it has been suggested that maladaptive social cognitions may be implicated in the maintenance of depression symptoms [21]. Although previous intervention studies have shown that social connectedness can be effectively improved through behavioural activation [21], educational programs [42], computer training [43], psychosocial education, or shared activities [44], maladaptive social cognitions have rarely been directly addressed in these interventions, even less so in older adult populations. Furthermore, interventions that target maladaptive social cognition have been shown to have larger effect sizes compared to interventions that enhance social support, improve social skills, or provide opportunities for social intervention [45]. Taken together, these findings underscore the potential benefits of enhancing personal motivation in facilitating greater participation, rather than just providing opportunities for social interactions.

In the current study, the intervention strategies in the enhanced CBT program are designed to increase meaningful social interactions through a combination of cognitive and behavioural techniques. Research indicates that a transdiagnostic CBT program for depression and anxiety may be effective in reducing loneliness in older adults, and that this reduction is likely to be facilitated by targeting shared underlying cognitive and behavioural mechanisms between loneliness, depression, and anxiety [20]. The current study will be testing out hypotheses around the application of CBT skills in addressing these underlying mechanisms. That is, participants in the enhanced CBT program will be encouraged to actively identify and challenge unhelpful or unrealistic cognitions related to social activities and interactions. This process is complemented by behavioural activation to encourage broader engagement in social activities, including scheduling regular activities with family and friends, participating in community group activities, and/or volunteering. Participants will also be encouraged to practise graded exposure to reduce avoidance of social interactions, as well as problem solving logistical barriers (e.g., cost and transportation). Furthermore, the enhanced CBT program will focus on improving assertiveness skills and conflict management to strengthen social relationships.

Our previous pilot data indicated that the enhanced CBT program, which incorporated a specific focus on increasing social participation in older adults, was associated with significant increases in social activities and overall quality of life, and marked reductions in symptoms and diagnoses of anxiety and depression. Furthermore, all changes were maintained at three-month follow-up and improvements in the social relationships’ component of quality of life suggested further improvement from post treatment to three-month follow-up. Participant feedback on the enhanced program was very positive. Importantly, when comparing effects from the enhanced CBT program of our pilot sample with the average results of standard CBT across our two larger RCTs [16, 17], the enhanced CBT program was associated with considerably large effects on both emotional distress and quality of life related to social relationships. Following these promising pilot data, the parallel group superiority randomised controlled trial design in this study will further evaluate the clinical efficacy of the enhanced CBT program for depressive and/or anxiety disorders in older adults, compared to the standard CBT program.

Outcomes from this study will provide evidence for an augmented psychosocial treatment for older adults suffering anxiety and/or depression. The treatment is supported by structured manuals, enabling dissemination. Bolstering meaningful social interactions in conjunction with exciting CBT skills is a novel approach to increase recovery rates for older adults suffering from depressive and anxiety disorders, as well as enhancing wellbeing and social participation in this age group, and thereby directly contributing to better economic outcomes. In addition to the empirical validation of a novel treatment, the current study will contribute to the current understanding of the role of social participation in older adult wellbeing.

### 3.1 Trial status

Recruitment commenced on 16^th^ July 2019. This study is expected to be completed by 31^st^ December 2022.

## Supporting information

S1 Checklist SPIRIT 2013 checklist: Recommended items to address in a clinical trial protocol and related documents*. (PDF)

S1 File (PDF)
