# Supplementary material for: Improving mental health and social participation outcomes in older adults with depression and anxiety: Study protocol for a randomised controlled trial
Source: PLoS One. 2022 Jun 27;17(6):e0269981. doi: 10.1371/journal.pone.0269981 (PMC9236237; doi:10.1371/journal.pone.0269981)
Supplement: S1 File — (PDF) [file pone.0269981.s002.pdf]

MACQUARIE UNIVERSITY

**Protocol Title:**
Improving Mental Health and Social Participation Outcomes in Older Adults with Depression and Anxiety

***NOTE**: This template is a guide. Sections that are not applicable (e.g. randomisation) can be deleted as required.*

Sections that are strongly recommended are: Hypothesis, Primary Objective, Inclusion and Exclusion criteria, Statistical Consideration for sample size, and Adverse Reporting if the study involves an intervention.

**Principal Investigator:**	Professor Viviana Wuthrich
**Contact Details:**	viviana.wuthrich@mq.edu.au; +61 2 9850 4866
**Protocol Number:**	1.0
**Protocol Authors:**	Viviana Wuthrich, Tess Jagiello, Denise Meuldijk, Jessamine Chen
**Protocol Version #:**	1.3
**Protocol Date:**	31.07.19
**Proprietary Notice** *(if applicable):*	

Signature: ________ ____

Date: ______________**Wednesday November 14th**___________________________

Ethics Statement:

The study will be conducted in accordance with the National Statement on Ethical Conduct in Human Research (2007), the CPMP/ICH Note for Guidance on Good Clinical Practice and consistent with the principles that have their origin in the Declaration of Helsinki. Compliance with these standards provides assurance that the rights, safety and well-being of trial participants are respected.

LIST OF INVESTIGATORS

Principal investigators

**Name:**	Viviana Wuthrich, Professor
Organisation:	Macquarie University
Address:	Centre for Emotional Health<br>Department of Psychology<br>Macquarie University<br>Sydney, NSW 2109
Telephone	02 9850 4866
Fax	02 9850 8062
Email	viviana.wuthrich@mq.edu.au
Role:	**Chief Investigator, CIA**

**Name:**	Ronald Rapee, Distinguished Professor
Organisation:	University of New South Wales
Address:	Centre for Emotional Health<br>Department of Psychology<br>Macquarie University<br>Sydney, NSW 2109
Telephone	02 9850 8032
Fax	02 9850 8062
Email	ron.rapee@mq.edu.au
Role:	**Chief Investigator, CIB**

**Name:**	Brian Draper, Professor
Organisation:	Prince of Wales Hospital, Randwick
Address:	Academic Department for Old Age Psychiatry,<br>Euroa Centre<br>Prince of Wales Hospital<br>Randwick, NSW, 2031
Telephone	02 9382 3753
Fax	02 9382 3762
Email	B.Draper@unsw.edu.au
Role:	**Chief Investigator, CIC**

**Name:**	Henry Brodaty, Scientia Professor
Organisation:	University of New South Wales
Address:	Centre of Healthy Brain Ageing<br>School of Psychiatry, Faculty of Medicine<br>University of New South Wales<br>Sydney, NSW 2052
Telephone	02 9385 2585
Fax	02 9385 2200
Email	h.brodaty@unsw.edu.au
Role:	**Chief Investigator, CID**

**Name:**	Henry Cutler, Dr
Organisation:	Macquarie University
Address:	Centre for the Health Economy<br>Ground Floor, Hearing Hub<br>University Drive, Macquarie University<br>Sydney, NSW 2109
Telephone	02 9850 2999
Fax	02 9850 9390
Email	henry.cutler@mq.edu.au
Role:	**Chief Investigator, CIE**

**Name:**	Lee-Fay Low, Associate Professor
Organisation:	The University of Sydney
Address:	Ageing, Work and Health Research Unit Faculty of Health Sciences<br>The University of Sydney<br>Lidcombe NSW 1825
Telephone	+61 2 9036 7368
Email	lee-fay.low@sydney.edu.au
Role:	**Chief Investigator, CIF**

**Name:**	Andrew Georgiou, Professor
Organisation:	Macquarie University
Address:	Centre for Health Systems and Safety Research<br>Australian Institute of Health Innovation<br>Macquarie University<br>Sydney, NSW 2109
Telephone	+61 2 9850 2424
Email	andrew.georgiou@mq.edu.au
Role:	**Chief Investigator, CIG**

**Name:**	Carly Johnco, Dr
Organisation:	Macquarie University
Address:	Centre for Emotional Health<br>Department of Psychology<br>Macquarie University<br>Sydney, NSW 2109
Telephone	02 9850 8053
Fax	02 9850 8032
Email	carly.johnco@mq.edu.au
Role:	**Chief Investigator, CIH**

**Name:**	Michael Jones, Professor
Organisation:	Macquarie University
Address:	Faculty of Human Sciences<br>Department of Psychology<br>Macquarie University<br>Sydney, NSW 2109
Telephone	02 9850 8601
Fax	02 9850 8032
Email	mike.jones@mq.edu.au
Role:	**Chief Investigator, CII**

**Name:**	Denise Meuldijk, Dr
Organisation:	Macquarie University
Address:	Centre for Emotional Health<br>Department of Psychology<br>Macquarie University<br>Sydney, NSW 2109
Telephone	02 9850 8655
Fax	02 9850 8032
Email	denise.meuldijk@mq.edu.au
Role:	**Postdoctoral Research Fellow**

**Name:**	Jessamine Chen, Dr
Organisation:	Macquarie University
Address:	Centre for Emotional Health<br>Department of Psychology<br>Macquarie University<br>Sydney, NSW 2109
Telephone	02 9850 9882
Fax	02 9850 8032
Email	jessamine.chen@mq.edu.au
Role:	**Postdoctoral Research Fellow**

**Name:**	Andrew Partington, Mr
Organisation:	Macquarie University
Address:	Centre for the Health Economy<br>Level 1, 3 Innovation Road<br>Macquarie University, NSW, 2109
Telephone	02 9850 1405
Fax	02 9850 9390
Email	andrew.partington@mq.edu.au
Role:	**Associate Investigator**

Contents

Protocol Title ... 1
Summary ... 3
1. BACKGROUND AND INTRODUCTION ... 5
1.1. Disease/Proposed Intervention Background ... 5
1.2. Rationale For Performing The Study ... 6
2. HYPOTHESIS ... 6
3. STUDY OBJECTIVES ... 7
3.1. Primary Objectives ... 7
3.2. Secondary Objectives ... 7
4. STUDY DESIGN ... 7
4.1. Design ... 7
4.2. Expected Participant Numbers ... 8
4.3. Duration Of The Study ... 8
4.4. Endpoints ... 8
4.5. Centres ... 9
5. STUDY PARTICIPANTS ... 9
5.1. Inclusion Criteria ... 9
5.2. Exclusion Criteria ... 9
6. STUDY PROCEDURES ... 10
6.1. Study Flow Chart ... 10
6.2. Investigation Plan ... 10
6.3. Study Procedure Risks ... 12
6.4. Participant Recruitment And Screening ... 13
6.5. Participant Enrolment ... 14
6.6. Information and Consent ... 14
6.7. Randomisation Procedure ... 14
6.8. End of Study Treatment/Withdrawal Procedure ... 14
6.9. Patient Withdrawal ... 15
7. OUTCOMES ... 15
7.1. Definition of Outcomes ... 15
8. STATISTICAL CONSIDERATIONS ... 18
8.1. Sample Size or Power Calculation ... 18
8.2. Detailed Analysis Plan ... 18
9. DATA COLLECTION ... 19

11. ETHICS 20
    11.1. Investigator Authorisation Procedure 20
    11.2. Patient Protection 20
12. SAFETY 20
    12.1. Adverse Event Reporting 20
    12.3. Data Safety and Monitoring Board (Dsmb) 21
    12.4. Early Termination 21
13. BLINDING AND UNBLINDING 21
14. CONFIDENTIALITY AND STORAGE AND ARCHIVING OF STUDY 22
15. TRIAL SPONSORSHIP AND FINANCING 22
16. INDEMNITY 22
17. REFERENCES 23
18. APPENDICES 26

Summary

**Study title:**	Improving Mental Health and Social Participation Outcomes in Older Adults with Depression and Anxiety
**Protocol version:**	Version 1.3, 31.07.19
**Objectives**	Primary objective: To evaluate the efficacy of an enhanced Cognitive Behavioral Therapy (CBT) program for treating anxiety and/or depression in older adults in comparison with standard CBT.<br><br>Secondary objective: To evaluate the cost-effectiveness of the enhanced CBT program for treating older adults with anxiety and/or depression in comparison with standard CBT.
**Study design**	Parallel group superiority randomised controlled trial (RCT) with two conditions: Enhanced CBT and Standard CBT.
**Planned sample size**	N = 172 (n = 86 for each condition)
**Selection criteria**	Inclusion criteria:<br>Older adults, aged 65 years and older, with a DSM-5 Anxiety and/or unipolar mood disorder as their primary (most interfering) presenting problem.<br><br>Exclusion criteria:<br>Insufficient English literary, psychosis or bipolar disorder; active suicidality, significant uncorrected hearing loss; likely moderate to severe dementia based on a standardized cognitive screener test.
**Study procedure**	Participants will be referred by their GP, or other health professionals, or can self-refer. Following referral, participants will be screened over the telephone by means of the study inclusion and exclusion criteria those deemed potentially suitable will be invited to complete the baseline measure including a structured diagnostic interview. Suitable participants will provide written informed consent to participate in the trial. Subsequently, participants will be randomly allocated to a condition (1) Enhanced CBT or 2) Standard CBT) and will make an appointment for their first session. Measures will be repeated at 14 weeks post baseline and at 12-month follow-up. Primary outcome is change on the Clinician's Severity Rating (CSR) of anxiety and

	depressive disorders. Secondary outcomes are changes in self-report measures. Cost effectiveness evaluation of the two conditions will be compared at 12-month follow-up. In addition, at the end of the study, participants will complete feedback questionnaires.
**Statistical considerations**	<u>Sample size calculation:</u> Given that we are comparing two active treatments the study has been conservatively powered to detect a difference between treatments at a medium effect (d=0.6). To provide power of .90, alpha = .05, two-tailed, we will need 172 participants (86 equally randomised to the two conditions). We estimate that we will need to assess 200 at baseline to obtain the final sample of 172 meeting all inclusion criteria.<br><br><u>Analysis plan:</u> To assess change on diagnostic severity (primary hypothesis) we will compare interventions at post-treatment (14 weeks post-baseline) and 12 month follow up (secondary hypothesis). Analysis will be a mixed model with random subject effect, fixed effects relating to group allocation (between-group), time (within-subject) and covariates. Interaction contrasts will be used if the time x group interaction reaches statistical significance but the main effect contrast will be used if not. Statistical interference will be based on the nonparametric bootstrap if the assumption of Normality is violated. Analyses will be "intent-to-treat", and multiple imputation will be used to estimate missing data. The same process will be used for the secondary hypotheses to compare the groups on all outcome measures.<br><br>An economic evaluation will be undertaken to measure the relative benefit in health outcomes and resource use for the enhanced intervention compared to standard CBT. The economic evaluation will be undertaken from a health care perspective. A separate economic evaluation developed from a societal perspective will include those resources from a health care system perspective and indirect resource use from broader changes in economic outcomes. The lifetime cost effectiveness of the intervention will be estimated using economic modelling techniques.
**Duration of the Study**	Three years (January 2019 – December 2021).

1. BACKGROUND AND INTRODUCTION

1.1. Disease/Proposed Intervention Background

Depression and anxiety in older populations are associated with poor physical health, disability, morbidity, increased costs of service use and medications as well as increased risk of cognitive decline (deBeurs et al., 2009; Draper, 2014; Norton et al., 2015; Wetherell et al., 2004). These disorders are furthermore associated with reduced social participation, poor social support, and increased feelings of loneliness and isolation (Douglas, Georgiou, & Westbrook, 2016; Hodgetts et al., 2017; Golden et al., 2009), which in turn increase risk for suicidal ideation, self-harm, suicide, and death (Almeida, Draper et al., 2012b; Oude Voshaar et al., 2016; Perissinotto et al., 2012). Consequently, the economic costs of these disorders for healthcare systems and society are high.

Recent systematic reviews and meta-analyses show that anxiety and depression can be successfully treated in older adults using psychological therapies. The strongest evidence base is for cognitive behavioural therapy (CBT) (Cuijpers et al., 2014; Gould et al., 2012a; 2012b). Moreover, since depression and anxiety commonly co-occur in older adults with almost 60% of older adults with depression also suffering an anxiety disorder and 30% of those with an anxiety disorder also suffering from depression (Almeida et al., 2012a), psychological programs that can effectively and efficiently treat both depression and anxiety are critically important for the mental health of older adults.

Our team developed the first transdiagnostic CBT program in the treatment of both depression and anxiety in older adults. In two randomised controlled trials (RCT) we demonstrated the efficacy of our program (*Ageing Wisely)* in reducing anxiety and depressive disorders in older adults (aged 60-88). The CBT program showed large reductions in diagnostic severity for anxiety disorders (Cohen's d=1.2) and unipolar depression (Cohen's d= 2.13) when compared to waitlist (Wuthrich & Rapee, 2013) and an active control (discussion group) (Cohen's d = 1.68 and 1.82, respectively) (Wuthrich et al., 2016). Improvements were maintained at least 6 months' following treatment (Cohen's d = 1.35-1.52). Just over 50% of participants were remitted from their main, presenting diagnosis and almost 40% were remitted from *all* mood and anxiety disorders at the end of treatment and up to 6 months after. Although these exciting results are among the strongest in the world, they underscore the significant proportion of older adults who maintain clinical levels of depression and anxiety following gold standard treatment. We are left looking for additional components that may boost the effects of current, best-practice CBT programs.

Social participation broadly refers to the degree to which a person participates in society, including through friendships, social groups, and volunteering. Older adults with symptoms of anxiety and depression have reduced social participation, poorer levels of perceived social support, and increased feelings of loneliness and isolation (Douglas et al., 2016; Golden et al., 1999; Hodgetts et al., 2017). The direction of causality between social interactions and depression is unclear, but is likely bi-directional (Hodgetts et al., 2017). That is, emotional distress most likely impedes social participation through its characteristics such as irritability, social withdrawal, anhedonia, and negative cognitive biases. At the same time a lack of quality social interactions is likely to directly increase feelings of worthlessness and rejection and thereby produce or maintain symptoms of anxiety, depression, and suicidal ideation. Douglas, Georgiou and Westbrook (2016) proposed that the impact of social

participation on health outcomes is likely to occur through increased social support and social cohesion; the more people participate the more likely they feel that they have people in the community to turn to and that people will help them. Hence, interventions that increase social participation through increased meaningful social contact, social group membership and volunteering should lead to increased quality of life, improved physical and mental health, as well as reduced mortality in older adults. By building stronger social supports and increased social contact, the maintenance of therapy outcomes is also likely to be enhanced.

Despite the strength of the evidence and theory, surprisingly few studies to date have examined the impact of targeting social factors on mental health outcomes in older adults. Some research has indicated the efficacy of CBT programs that specifically target loneliness (mean Cohen's d = .6; Cacioppo et al., 2015). But no studies have evaluated the effect of targeting a broader concept of social participation or integrating this focus into standard CBT programs in the management of emotional disorders. Given the strong bi-directional relationships between social participation and emotional distress, it is likely that standard treatments for anxiety and depression in older adults will benefit from a direct focus on social participation. As anxiety, depression and lack of social stimulation are large risk factors for the development of cognitive decline in older adults, we will also examine changes in cognitive abilities over time.

1.2. Rationale For Performing The Study

The main aim of this project is to evaluate the efficacy of delivering a psychosocial intervention to treat emotional symptoms whereby increasing social participation (enhanced CBT) in older adults with anxiety and/or depression. We will examine the effectiveness and cost-effectiveness of this enhanced CBT program compared to the current best practice transdiagnostic CBT program for depressed and anxious older adults (Wuthrich et al., 2016; Wuthrich & Rapee, 2013). The enhanced CBT program has the potential to increase recovery rates for older adults suffering from mood an anxiety disorders, enhance wellbeing and social participation in this age group and will directly contribute to beneficial economic outcomes. The results of this study will contribute to the improvement of the efficacy of current existing programs to manage anxiety and depression and will enhance the development of new (cost)-effective treatment options for these disorders in older adults. In addition, we will examine moderators of treatment response, and changes in cognitive ability over time.

2. HYPOTHESIS

1. We predict that the enhanced program will lead to significantly greater reductions on our primary outcome, diagnostic severity of all anxiety and unipolar mood disorders, compared to standard CBT program. immediately post-treatment (Primary Hypothesis)

2. We predict that the enhanced program will lead to greater reductions in diagnostic severity of all anxiety and unipolar disorders compared to standard CBT at 12-month follow-up (Secondary Hypothesis).

3. We similarly predict that the enhanced program will show significantly better outcomes than standard CBT on a range of related measures including self-reported depression,

anxiety, suicidal ideation, loneliness, and quality of life immediately post-treatment and at 12 month follow up (Secondary Hypotheses), and cognitive outcomes at 12 month follow up.

4. We predict that the enhanced CBT intervention will show greater cost-efficacy at 12-month follow-up compared with standard CBT (Secondary Hypothesis).

5. We predict treatment outcomes will be moderated by the presence of personality disorders, demographics, symptom severity and baseline cognitive ability.

3. STUDY OBJECTIVES

3.1. Primary Objectives

To evaluate the efficacy of the enhanced CBT program for treating older adults with depression and/or anxiety in comparison to current "best practice" transdiagnostic CBT (standard CBT).

3.2. Secondary Objectives

To evaluate the cost-effectiveness of the enhanced CBT program for treating older adults with depression and/or anxiety in comparison to standard CBT. In addition, to understand the moderators of treatment outcomes, and impact on cognitive outcomes. Additionally, patient and therapist satisfaction with the enhanced CBT program will also be explored.

4. STUDY DESIGN

4.1. Design

The main study design uses a parallel group superiority randomised controlled trial design to evaluate the clinical and cost-effectiveness of an enhanced CBT program for depressive and/or anxiety disorders in older adults, compared to standard CBT.). After baseline examination suitable participants will be randomised to either the 1) Standard CBT or 2). Enhanced CBT treatment condition.

Standard CBT.

The standard program will comprise our empirically validated CBT program for older age anxiety and depression, Ageing Wisely. Ageing Wisely consists of 11, weekly sessions to teach practical skills to help manage anxiety and depression including: goal setting, activity scheduling, problem solving, graded exposure, cognitive restructuring, assertiveness skills, and sleep hygiene. For the current trial we will add one treatment session to equate contact hours between treatment conditions. Homework exercises are a critical component that assists skills to be generalised and maintained.

Enhanced CBT.

The enhanced CBT program comprises 12 weekly sessions, teaching the same CBT skills as the standard Ageing Wisely program, but with a stronger focus on bolstering social participation and connections within those skills.

In both treatment conditions, all sessions will be run by clinical psychologists and intern psychologists trained in the delivering of the treatment protocols. To control for therapist differences, all therapists will be trained in and will conduct both treatments (allocated randomly). Supervision will be provided by CIs Johnco and postdoctoral fellows managing the trial with treatment adherence and differentiation between conditions as a core focus. All therapy sessions will be recorded, and a random 25% will be rated by an independent expert unaware of the study hypotheses for fidelity to the therapeutic model using a codebook and form based on Waltz et al. (1993).

The effectiveness of the programs will be established using mixed model analysis to compare the differences in clinical diagnostic severity (established by clinicians blind to treatment allocation) and scores on self-report measures at pre-treatment, post-treatment, and at a 12-month follow up period. Treatment integrity and adherence checks will be conducted to ensure treatment conditions were accurately delivered. In addition, an economic evaluation will be undertaken to measure the relative benefit in health outcomes and resource use for the enhanced intervention compared to standard CBT. Moderation analyses will examine the impact of moderators on treatment outcomes.

4.2. Expected Participant Numbers

We expect to recruit N = 172 at the Macquarie University Centre for Emotional Health Clinic, with participants equally randomised to either the enhanced CBT program or the standard CBT program.

4.3. Duration Of The Study

Data collection will commence when ethics is granted (ideally late 2018). It is expected that the study participants will be recruited until mid 2020. The study is anticipated to be completed by the end of 2021.

4.4. Endpoints

Primary Endpoint: The primary endpoint is immediately post-treatment.

Secondary Endpoints: The secondary endpoint is 12 months post baseline.

4.5. Centres

The trial will run through the Centre for Emotional Health Clinic at Macquarie University. The Macquarie University Centre for Emotional Health Clinic has developed a growing reputation for the treatment of older age anxiety and depression over the past six years, and has a regular referral flow of patients. We expect to recruit 172 participants at this site.

5. STUDY PARTICIPANTS

5.1. Inclusion Criteria

Participants will be referred to the Macquarie University Centre for Emotional Health Clinic t by their GP, primary health network, other health professional or health organization (e.g. aged care, hospital, residential aged care), or can self- refer. In addition, the study will be advertised broadly to the community and the GPs in the local areas of the sites, using brochures, media and newspaper stories and advertisements, and advertised through Beyond Blue's network of GPs in the local area (see Section Participant Recruitment and Screening).

Inclusion criteria: Aged ≥ 65 years; anxiety and/or unipolar depression is the main interfering problem according to the DMS 5 criteria assessed by a semi-structured diagnostic interview.

5.2. Exclusion Criteria

Exclusion criteria will be English language illiteracy, psychosis or bipolar disorder, active suicidality, significant uncorrected hearing loss and likely moderate to severe dementia based on a standardized cognitive screener test (i.e. the scores on the six-item Cognitive impairment Screener). Participants currently using (psychotropic) medications will not be excluded, but participants will need to be stabilised on medications for 1 month before baseline assessments. All medication changes will be noted at each assessment and controlled for in analyses.

6. STUDY PROCEDURES

6.1. Study Flow Chart

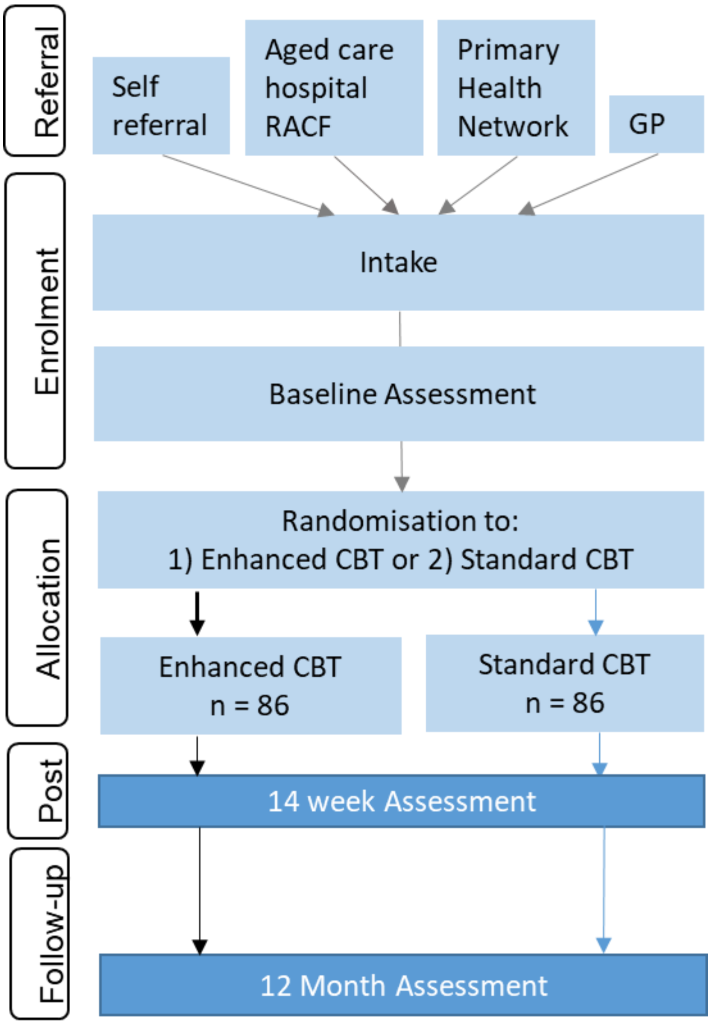

6.2. Investigation Plan

	**Enrolment**	**Timepoint 1**	**Timepoint 2**	**Timepoint 3**
**Study Procedures**	**Intake / Screening**	**Baseline Assessment**	**Post - treatment 14 weeks Assessment**	**Follow- Up 12 Month Assessment**
Inclusion / Exclusion criteria	√√	√√		
Informed Consent	√√			
Randomisation		√√		
Measures[a]				

Basic Demographics[b]	√√			
Detailed Demographics		√√		√√
Cognitive Impairment Screener – 6 item	√√			
Cognitive Assessment Battery		√√		√√
Anxiety Disorder Interview Schedule) – version 5 (ADIS-5)		√√		
Clinician's Severity Rating (CSR) on ADIS		√√	√√	√√
Geriatric Anxiety Inventory (GAI) – 20 item		√√	√√	√√
Geriatric Anxiety Scale (GAS) – 30 item		√√	√√	√√
Geriatric Depression Scale (GDS) – 30 item		√√	√√	√√
Depressive Symptoms Inventory – Suicide Subscale		√√	√√	√√
De Jong Gierveld Loneliness Scales – 11 item		√√	√√	√√
Lubben Social Network Scale (LSN)		√√	√√	√√
Big Five Inventory (BFI)		√√	√√	√√
Davos Assessment of the Cognitive Biases Scale (DACOBS)		√√	√√	√√
The Personality Inventory for DSM- 5 (PID-5-BF)		√√	√√	√√
The Cognitive Distortions Scale (CDS)		√√	√√	√√
The Working Alliance Inventory (WAI)		√√	√√	√√
Bille- Brahe Social Support scale		√√	√√	√√
Societal Participation Questionnaire (SPQ)		√√	√√	√√
Australian Community Participation Questionnaire short form (ACPQ)		√√	√√	√√
Australian Quality of Life (AQoL-8D)		√√	√√	√√
Use of Service Questionnaire		√√	√√	√√

Consumer Questionnaire			√√	
iMTA Productivity Cost Questionnaire				√√
Health use Data Extraction CHeReL		√√	√√	√√

[a] Measures will be administered over the phone or online or paper based (participant's choice)

[b] Basic demographics will be sourced to compare group differences in participants who agree to participate versus those that do not agree to participate, and will be extracted from client records. Basic demographics will be age, gender, and mean CSR at baseline.

6.3. Study Procedure Risks

This study does not pose any risk for social, financial, or physical harm.

1. *Emotional distress* – There is a small risk that participants may find some personal assessment questions emotionally distressing. However, the questions asked are not beyond the scope of those asked in a regular intake procedures, assessment and therapy sessions. In addition, it is likely the benefit of the research justifies the risks of harm or discomfort to participants. The measures will be administered by trained research assistants and intake officers, registered and intern psychologists supervised by a Clinical Psychologist.
2. *Risk of self-harm* – As this study is actively recruiting participants who are distressed there is a risk that an individual may engage in self-harm including making a suicide attempt. The team are experienced in working with older distress individuals and will follow clinic protocols for managing risk. This includes regular and active screening of self-harm risk including intent, access to means, and previous attempts. In the case that risk is identified study protocols will be followed which include escorting the individual to a safe place for further psychiatric assessment (such as the University Medical Centre or calling the local crisis team), informing relevant family members and GP of the risk, and further of monitoring the risk).
3. *Burden of completing measures* - As part of the study, participants will be asked to complete a semi-structured interview, complete cognitive assessments and answer questionnaires. Our team have run two similar clinical trials in older adults with anxiety AND depression using a very similar assessment protocol. The face to face assessments are usually conducted in two segments – 1.5 hours for the cognitive assessment, followed by a lunch break, and then 1.5 hours for the clinical interview. The baseline measurement is the longest assessment, with the post- and follow- up assessments of the clinical interview usually taking only 30 minutes. In addition, participants will complete self-report questionnaires on mood, wellbeing and medical service use which will take approximately 30 minutes that they will do at home in their own time.
4. *Privacy* - participants will be asked to release some private information to researchers. This information will be stored under alpha numeric codes on a secure university server in password protected files and so the risk of being identifiable is very remote. The research team have significant experience running clinical trials and managing private information.

5. *Coercion*- participants might feel pressured to participate in the research. Coercion will be minimized by training all staff involved in recruitment or the study in anyway in the appropriate ways of providing information about the study, gaining informed consent, notifying participants about the voluntary nature of the research and right to withdraw consent at any time without consequence. Participants will be given time to make decisions about agreeing to participate in the study.

6.4. Participant Recruitment And Screening

Participants will be recruited from GPs, primary health network, other health professional or health organisations (e.g. aged care, hospital, residential ages care) or can self-refer. In addition, the study will be advertised broadly to the community and the GPs in the local areas of the sites, using brochures, media and newspaper stories and advertisements, and through Beyond Blue's professional networks. The GPs in the local area of the sites will be informed of the study and given information about how to improve detection and screening of anxiety and depression in their elderly patients.

The Macquarie University Centre for Emotional Health Clinic has built a strong reputation for psychological treatments and a wide referral base over the past 15 years. The Macquarie University Centre for Emotional Health Clinic has developed a growing reputation for the treatment of older age anxiety and depression over the past six years, and has a regular referral flow of patients. We expect to recruit 172 participants at this site.

Following referral, participants will be screened over the telephone. Intake officers will assess likely suitability for the trial based on inclusion and exclusion criteria (as outlined in this application). Older adults who are likely to meet the criteria will be sent participant information and consent forms. Intake officers will follow-up with patients the next day or at a time convenient to the participant, to review understanding of the information and consent form and to answer any questions. Patients who are interested in participating will be asked to provide written informed consent. They will then make an appointment to complete full baseline measures according to the study design, including a semi-structured diagnostic interview (administered by a clinical registrar at Macquarie University), to finalise suitability. After completion of this baseline assessment, participants' randomization by the research team takes place (see Section Randomisation Procedure) and an appointment will be scheduled for their first treatment session.

If the older adult is judged to be ineligible for enrolment in the current study, the primary reason of ineligibility will be documented and the older adult will be considered as unsuitable to participate in the trial. Participants who do not consent or do not meet eligibility criteria will be offered alternative services at the Centre for Emotional Health clinic as appropriate and/or referred to alternative services in the local area and/or to their GP.

See Appendices for copies of advertising flyer, information brochure, participation information sheet, and consent form.

6.5. Participant Enrolment

After obtaining the participants' informed consent and verifying that the participant fulfils all the inclusion and none of the exclusion criteria, they will be allocated a de-identified alpha-numeric code allocated by the Centre for Emotional Health clinic and randomised to the treatment condition.

6.6. Information and Consent

Informed consent will be obtained from all participants. Intake officers will notify the older adult about the study and send participants (via email or mail) an information and consent form outlining what exactly is involved (see Appendix A). Written informed consent will be obtained from all participants. Consent will be collected prior to the baseline assessment. See Appendix A for written consent procedure.

6.7. Randomisation Procedure

Suitable participants will provide informed consent and will be subsequently randomised to receive enhanced CBT or standard CBT.

The randomisation schedule will be generated using a computerised randomiser (www.randomizer.org) and placed into consecutively numbered sealed envelopes by Faculty statistician independent of the research team).

6.8. End of Study Treatment/Withdrawal Procedure

End of Treatment

The primary endpoint for this study is the 14 weeks post treatment assessment. The secondary endpoint for this study takes place 12 months after baseline (12 month Follow Up assessment). At the secondary endpoint, all participants will be encouraged to continue to use the skills they have learned from the treatment.

Continuation of therapy

Participants who are still reporting clinically significant impairment at the secondary endpoint will be provided with additional services as per usual practice at these sites, or if appropriate, can be referred to services elsewhere.

6.9. Patient Withdrawal

Reasons for withdrawal

Participants can withdraw from the study at any time and still receive the treatment without consequence. Participants are unlikely to withdraw from the study, but might choose to withdraw from treatment (stop attending appointments). The most likely reason for participant withdrawing from treatment it that they are feeling better and no longer require treatment. If participants withdraw from treatment, we will still ask them for follow up data at the assessment points. They will be free to withdraw from the study and not provide this data. Wherever possible, information about their decision to withdraw from the study will be collected in a non-threatening way, to comply with CONSORT recommendations and to facilitate the correct reporting of the impact of adverse events on treatment completion.

In the rare event that a participant ends up in crisis due to external circumstances that prevents them from continuing in treatment, or if an alternative treatment becomes indicated (e.g. pharmacology, hospital admission), participants will continue to be monitored throughout the study and complete follow up assessments (as practical) and the failure to complete treatment taken into consideration in the analysis and reporting of the results of the trial.

Handling withdrawal and losses to follow- up

If a participant wishes to withdraw from the research once it has started, they can do so at any time without impacting their future treatment at any of the participating sites. Wherever possible, information about their decision to withdraw from the study will be collected in a non-threatening way, to comply with CONSORT recommendations and to facilitate the correct reporting of the impact of adverse events on treatment completion. Verbal or written withdrawal of participation will be accepted. See the statistical handling of missing data below.

7. OUTCOMES

7.1. Definition of Outcomes

The aim of this study is to evaluate the efficacy and cost-effectiveness of an enhanced CBT program in the treatment of anxious and/or depressed older adults, relative to current 'best practice' transdiagnostic CBT (standard CBT).

Assessments

The study has a longitudinal design conducted over 12 months with three assessment points in both the enhanced CBT and standard CBT condition. Outcomes will be collected at baseline, 14 weeks, and 12 month follow-up.

1. Baseline assessment – (Prior to program entry and completed during intake)
2. Post-treatment 14 weeks assessment – (14 weeks from baseline assessment)
3. Follow-up 12 months assessment – (12 months from start of therapy)

Outcomes

1. Primary outcome

The primary outcome is the Clinician's Severity Rating (CSR) of anxiety and depressive disorders based on the *Anxiety Disorders Interview Schedule 5th edition (ADIS -5)* **post-treatment**. *The ADIS -5* is a semi-structured interview for diagnosing psychiatric conditions according to DSM -5 criteria. This interview will be administered by clinical psychologist and registrars who will be trained to criterion in its administration, and receive weekly supervision to assist with diagnostic decisions.

2. Secondary Outcomes

a. Clinician's Severity Rating (CSR) of anxiety and depressive disorders based on the Anxiety Disorders Interview Schedule 5th edition (ADIS -5) **at 12 month follow-up**.

b. Changes in Symptomatology on the following measures:

Geriatric Anxiety Inventory (GAI), is a 20 item self-report measure of anxiety, with good internal consistency, test-retest reliability and concurrent validity (Pachana et al., 2007).

Geriatric Anxiety Scale (GAS) is a 30-item self-report measure used to assess anxiety symptoms among older adults *(*Segal et al., 2010).

The *Geriatric Depression Scale (GDS),* is a 30 item self-report measure of depression for use in older adult populations (Yesavage, et al., 1983).

The *Depressive Symptoms Inventory – Suicide Subscale* (Joiner Jnr, 2002), is a 4 item measure of suicidal ideation.

c. Changes in Social Participation

De Jong Gierveld Loneliness Scales (De Jong Gierveld & Tilburg, 2006) measures feelings of loneliness and perceived social isolation with 11 items.

The Lubben Social Network Scale (LSNS) is a commonly used tool to assess social connections in older adult populations. The 18 item scale assesses the frequency and quality of family, friendship and neighbour support.

The Bille- Brahe Social Support scale (8 –item), a short scale to measure social support and social integration (Bille-Brahe, 1985).

d. Measures of Cost Effectiveness

The *Australian Quality of Life* (AQoL-8D), measures quality of life and health outcomes across eight domains (independent living, relationships, mental health, coping, pain, senses, self-worth and happiness) and can be used to calculate QALYs (see below).

Health Resource Use, data will be sourced using a purpose built medical cost questionnaire, supplemented with linked data from the NSW Centre for Health Record Linkage (CHeReL) on Admitted Patient data, Emergency Department data, and Mental Health Ambulatory data, along with Medicare Benefit Schedule (MBS) and Pharmaceutical Benefit Schedule (PBS) data.

The *iMTA Productivity Cost Questionnaire* (Bouwmans et al., 2015), is a standardised instrument to measure and value health-related productivity losses including presenteeism and absenteeism in paid and unpaid work.

Use of Service questionnaire, constructed by the CIs to assess use of health and community care services in the last 6 months. This includes hospital services, medical services, allied health services, community based services, and diagnostic services.

A Societal Participation Questionnaire (SPQ), will be constructed by the CIs to assess frequency and types of social activity (including volunteering, childcare assistance, social events).

The Australian Community Participation Questionnaire short form (ACPQ), is a 15 item measure of frequency of participation in 7 different social activities (i.e. contact with extended family, friends and neighbours, contact with friends and family, attending organised community events, volunteering and political protest).

e. Moderators of Treatment

The Big Five Inventory (BFI; John & Srivastava, 1999) is a 44 item self-report inventory designed to measure the Big Five dimensions. Each of the factors is then further divided into personality facets.

The *Davos Assessment of the Cognitive Biases Scale* (DACOBS; van der Gaag et al. 2013), a 42 –item self-report tool for a broad assessment of different kinds a cognitive distortions.

The *Personality Inventory for DSM-5—Brief Form (PID-5-BF)—*Adult is a 25-item self-rated personality trait assessment scale for adults age 18 and older. It assesses 5 personality trait domains including negative affect, detachment, antagonism, disinhibition, and psychoticism, with each trait domain consisting of 5 items (Krueger et al. 2013).

The cognitive Distortions Scale (CDS; Covin et al., 2011) is a 20 item scale that measures the tendency to make ten cognitive distortions including mindreading, catastrophising, all or nothing thinking. It has sound psychometric properties with clinically relevant measures and appears relevant for clinical and research populations.

The Working Alliance Inventory (WAI-Short-Form: Paap & Dijkstra, 2007) is a 36 item scale to measure the therapeutic alliance between a therapist and a client. There is a therapist and a client form. It is based on Bordin's theory of working alliance and measures the agreement between the therapist and the client on goals of therapy and well as tasks to achieve these goals. This scale has sound psychometric properties with other measures in this field.

f. Cognitive Assessment Battery.

Neuropsychological Assessment will assess attention/orientation, verbal fluency, memory, language and spatial abilities, using a neuropsychological battery consisting of the: Mini-Mental State Examination, Logical Memory I & II, Benton Visual Retention Test, Verbal Fluency (FAS and animals), Digit Symbol, Boston Naming Test, Rey Auditory Visual Learning Test, Block Design, National Adult Reading Test, Trail Making Test, and Grooved Pegboard. This will take less than 90 minutes.

g. Consumer Questionnaire

A purpose built questionnaire with open and closed questions will be used to gather information on consumers' experiences, benefits, barriers and dislikes about the psychosocial interventions.

8. STATISTICAL CONSIDERATIONS

8.1. Sample Size or Power Calculation

Based on review of the available literature and our pilot study results comparing this enhanced CBT program to an active control group, we expect the enhanced CBT program to produce larger effects on the treatment outcomes t in the range of Cohen's d ~ 0.6 – 1.0. However, given that we are comparing two active treatments, we will conservatively power this study to detect a difference between treatments at a medium effect (d=0.5).

Therefore based on a moderate effect of d=0.5, with power at 90% and, alpha at0.05 (2 sided) we will need at least 172 subjects (86 equally randomised to each condition) in total. With the expectation of 15% of participants to be found to be unsuitable, at least 200 participants will be needed.

8.2. Detailed Analysis Plan

Efficacy Evaluation:

Analyses will be conducted using intention-to-treat and as such all participants will be analysed in the group to which they were randomised. Multiple imputation will be used to manage missing data. The effectiveness of the programs will be established using mixed model analysis to compare the differences in clinical diagnostic severity (established by clinicians blind to treatment allocation) and scores on self-report measures at pre-treatment, post-treatment, and at a 12-month follow up period. Analysis will be a mixed model with random subject effect, fixed effects relating to group allocation (between-group), time (within-subject) and covariates. Interaction contrasts will be used if the time x group interaction reaches statistical significance but the main effect contrast will be used if not. Statistical inference will be based on the nonparametric bootstrap if the assumption of Normality is violated.. The same process will be used for the secondary hypotheses to compare the groups on all outcome measures.

Economic Evaluation

An economic evaluation will be undertaken from both the healthcare and societal perspective to measure the relative benefit in health outcomes and resource use for the enhanced intervention compared to standard CBT.

Health outcomes will be evaluated using Quality Adjusted Life Years (QALYs) derived from scores on the AQoL-8D. Resource use per patient in the enhanced CBT intervention versus the cost per patient in the standard CBT group will be compared and will include acute care,

primary care, mental health care, community care and medication use. Productivity (employment, absenteeism and presenteeism) will be captured using the validated iMTA Productivity Cost Questionnaire (iPCQ), while other valuable activities such as volunteering and caring will be captured using the purpose built societal participation questionnaire. Cost utility analysis would be undertaken, comparing differences in QALYs with differences in resource use, and presented using an incremental cost effectiveness ratio (ICER). This will provide a basis to make judgements on whether the enhanced CBT intervention is cost effective, by comparing the ICER to the federal government's willingness to pay threshold for QALYs (e.g., $50,000 per QALY).

The lifetime cost effectiveness of the intervention will be estimated using economic modelling techniques, such as Markov modelling. Uncertainty around the ICER and lifetime cost effectiveness results will be explored using deterministic sensitivity analysis (varying model parameter values manually) and probabilistic sensitivity analysis (drawing model parameter values simultaneously from a priori–defined probability distributions using Monte Carlo simulation methods). Threshold analysis will be used to estimate levels of uncertain parameters where the enhanced intervention becomes cost effective. This will reduce uncertainty for decision makers when deciding on whether to fund the enhanced intervention as an ongoing program, and roll it out into other health services.

Mediation Analyses:

Mediation will be evaluated using path models. Of key interest are the total effect (sum of all paths between clinical severity rating at baseline and 12 month follow-up), the direct path between clinical severity rating at baseline and 12 month follow-up, and the indirect path (total-direct) via key demographic variables, personality and social participation factors. Statistical inference will be based on the nonparametric bootstrap if the assumption of Normality is violated.

9. DATA COLLECTION

Outcomes will be collected by self-report measures (collected online, via pen-and-paper or over the telephone at the participant's choice) and a semi- structured diagnostic interview (ADIS-5) at baseline, 14 weeks, and at 12 month follow-up by assessors blinded to treatment allocation. See Section 14.CONFIDENTIALITY AND STORAGE AND ARCHIVING OF STUDY for detailed information about Data Storage.

All collected participant data will be allocated a de-identifiable alpha numeric code so that confidentiality is maintained during the study, and when the data is stored. See Section 6. STUDY PROCEDURES and Section 7. Outcomes for detailed information about the data collection and outcomes.

10. QUALITY CONTROL AND ASSURANCE

Reliability checks will be conducted by a qualified independent rater (research psychologist or research assistant). In order to satisfy treatment integrity checks, for all participants who consented to recording of therapy sessions, all therapy sessions will be recorded and 20%

randomly selected for reliability coding by an independent scorer for treatment integrity and adherence coding. In addition, clinicians conducting assessment and therapy will undergo weekly supervision with a clinical psychologist managing the study.

11. ETHICS

11.1. Investigator Authorisation Procedure

Ethics approval will be sought from the Macquarie University Human Research Ethics Committee (HREC)- Medical Science. Furthermore, after ethics approval, the trial will be registered on the Australian New Zealand Clinical Trials Registry (ANZCTR) prior to enrolment of the first participant.

11.2. Patient Protection

The study will be completed in accordance with the guidelines set out in the National Statement on Ethical Conduct in Human Research (2007) (the National Statement) and the CPMP/ICH Note for Guidance on Good Clinical Practice and any other relevant legislation/guidelines.

The results of the study will be presented at national and international conferences, and submitted for publication in peer-reviewed journals. In these instances, only de-identified data will be used, and only averages will be reported. No individually identifying information will be reported.

12. SAFETY

12.1. Adverse Event Reporting

This trial does NOT involve a therapeutic product and so no adverse events are anticipated. Given the participants are emotionally distressed, it is unlikely but possible that a participant may commit suicide, engage in self-harm, or require immediate psychiatric assessment or institutionalization for safety purposes.

To minimise any risk of harm, suicide intent is screened at intake and baseline, and as needed throughout the trial. Any unforeseen events or clinical complications that arise will be reported by clinicians and the research team during regular project supervision and to the chief investigator. As noted in 6.3 above, the team are experienced in working with older distress individuals and will follow clinic protocols for managing risk. This includes regular and active screening of self-harm risk including intent, access to means, and previous attempts. In the case that risk is identified study protocols will be followed which include escorting the individual to a safe place for further psychiatric assessment (such as the University Medical Centre or calling the local crisis team), informing relevant family members and GP of the risk, and further of monitoring the risk). The chief investigators A/Professor Viviana Wuthrich and Professor Ron Rapee will make decisions in discussion

with other CIs about any needed course of action. All adverse events will be documented and the Independent Data Safety Board (DSMB) will be informed. All serious adverse events will be reported to the HRECs within 72 hours. The reports will be followed by a detailed written report. Follow-up reports will identify a participant by their unique code rather than by their name.

12.3. Data Safety and Monitoring Board (Dsmb)

A data safety and monitoring board (DSMB) services in an independent capacity and provides their expertise and recommendations to the research group. The data safety and monitoring board consists of an independent group of experts. The primary responsibilities of the DSMB are to 1) periodically review and evaluate the accumulated study data for participant safety, study conduct and progress, and, when appropriate, efficacy, and 2) make recommendations to the research group concerning the continuation, modification, or termination of the trial.

An advisory committee including a geriatric psychiatrist, clinical psychologist and two consumer advocates is being finalised and will meet prior to, and 6 monthly, for the trial duration. The CIs will report to the independent DSMB on all adverse events, as well as to the relevant Ethics committee.

12.4. Early Termination

There are no foreseeable reasons for early termination of the study. In the unlikely case of early termination, it is the responsibility of A/Professor Wuthrich to inform all the involved staff about the management of study termination in collaboration with the involved Ethics Committee.

An earlier end of the clinical trial, which is not based on grounds of safety, but on other grounds, such as faster recruitment, than anticipated, is not considered as ‘early termination’.

13. BLINDING AND UNBLINDING

A Faculty Statistician (independent of the research team) will create the randomisation schedule to allocate individuals to condition. Suitable participants will be randomly allocated by the Research Assistant off site. Due to the nature of randomisation by site, clinicians and participants will not be blinded to condition.

Clinical diagnostic severity will be assessed by clinicians blind to treatment allocation. This is critical to examine the effectiveness of the programs.

14. CONFIDENTIALITY AND STORAGE AND ARCHIVING OF STUDY

Data confidentiality

All participant data will be allocated a de-identifiable alpha- numeric code so that confidentiality is maintained during the study, and when the data is stored.

Data Storage

All data will be stored via a de-identifiable alpha- numeric code. Paper copies of questionnaires will be entered into the electronic database (and then archived). An electronic record linking the alpha- numeric IDs with patient identifying information (such as Medicare number) will be kept in a separate password protected electronic file stored by the chief investigator and project manager. The electronic database will be stored in online data storage repositories indefinitely at Macquarie University in a password protected folder. Only CIs and project manager (postdoctoral research fellow) and research assistant will have access to this de-identified data.

After data collection is complete, de-identified data may be shared with other researchers, used in subsequent studies, or stored in online data repositories. Sharing of the de-identified data will help to maximise the benefits that may result from this study.

Study Record Retention

Study records will be retained for a minimum of 15 years after the last publication arising from the study.

15. TRIAL SPONSORSHIP AND FINANCING

This study is co-funded by NHMRC and Beyond Blue. The investigators declare no conflict of interest. The fund awarded can be used to cover the direct costs of the research proposal (recruitment, treatment, dissemination of results).

16. INDEMNITY

16.1. Compensation

Reasonable precautions against harms are being taken, including details of any hazards noted in the risk assessment, and action taken to reduce or eliminate risk. The administering institution agrees to follow the Medicines Australia Guidelines for Compensation for Injury Resulting from Participation a Company-Sponsored Clinical Trial.

The Macquarie University Code for the Responsible Conduct of Research (Macquarie Code) outlines standards of responsible and ethical conduct expected of all persons engaged in research under the auspices of Macquarie University. The University has developed this Code

to meet the standards set out in the Australian Code for the Responsible Conduct of Research (2007). The administering institution agrees to follow the Medicines Australia Guidelines for Compensation for Injury Resulting from Participation a Company-Sponsored Clinical Trial.

17. REFERENCES

ABS 2015. Disability, ageing and carers, Australia: summary of findings, 2012. ABS cat. no. 44300DO003_2012. Canberra: ABS

ABS, 2013. Causes of death, Australia, 2013. ABS cat no 3303.0. Canberra, ABS.

Almeida OP, Draper B, Pirkis J, Snowdon J, Lautenschlager NT, Bryne G,...Pfaff JJ. (2012a). Anxiety, depression, and comorbid anxiety and depression: risk factors and outcome over two years. *International Psychogeriatrics*, 24,1622-1632.

Almeida OP, Draper B, Snowdon J, Lautenschlager NT, Pirkis J, Bryne G,...Pfaff JJ. (2012b). Factors associated with suicidal thoughts in a large community study of older adults. *British Journal Psychiatry*, 201, 466-472.

Australian Institute of Health of Wellfare (2011), Trends in hospitalised injury, Australia 1999-00 to 2010-11, AIHW.

Berry HL, & Welsh JA. (2010). Social capital and health in Australia: An overview from the household, income and labour dynamics in Australia survey. *Social Science & Medicine*, 70, 588-596.

Blanc, ALL, Bruce, L, Heimberg, RG, Hope, DA, Blanco, C, Schneier, FR, Liebowitz, M, (2014). Evaluation of the Psychometric Properties of Two Short Forms of the Social Interaction Anxiety Scale and the Social Phobia Scale. *Assessment*. 21 (3): 312- 323.

Bouwmans C, Krol M, Severens H, Koopmanschap M, Brouwer W, Hakkaart-van Roijen L 2015. The iMTA Productivity Cost Questionnaire: A standardised instruement for measuring and valuing health-related productivity losses. *Value in Health*, 753-758.

Bille - Brahe U., Hansen W., Kolmos L. & Wang AG. (1985), Attempted suicide in Denmark. *Acta Psychiatrica Scandinavica*, 71, 217-226, http://doi:10.1111/j.1600-0447.1985.tb01277.x

Cacioppo S, Grippo A J, London S, Goossens L, & Cacioppo JT. (2015). Loneliness: Clinical Import and Interventions, *Perspectives on Psychological Sciences*, 10 (2), 238-249.

Callahan CM, Unverzagt FW, Perkins A J, & Hendrie HC. (2002). Six-item screener to identify cognitive impairment among potential subjects for clinical research. *Medical Care,* 40 (9), 771-81.

Cohen A, Gilman SE, Houck PR, Szanto K, Reynolds CF 2009. Socioeconomic status and anxiety as predictors of antidepressant treatment response and suicidal ideation in older adults. *Social Psychiatry and Psychiatric Epidemiology,*44, 272–277.

Covin R, Dozois DJA, Ogniewicz A & Seeds PM. (2011). Measuring Cognitive Errors: Initial Development of the Cognitive Distortions Scale (CDS), *International Journal of Cognitive Therapy,* 4, Special Section: Hoarding, pp. 297-322, https://doi.org/10.1521/ijct.2011.4.3.297

Cuijpers P, Karyotaki E, Pot AM, Park M, & Reynolds CF. Managing depression in older age: psychological interventions. 2014 *Maturitas*, 79(2), 160–169.

de Beurs E, Beekman ATF, van Balkom AJLM, Deeg DJH, van Dyck R, & van Tilburg W. (2009). Consequences of anxiety in older persons: its effect on disability, wellbeing and use of health services. *Psychological Medicine*, 29, 583-593.

de Jong Gierveld J, Tilburg TG. 1999. Manual of the loneliness scale. Amsterdam: VU University.

Douglas H, Georgiou A, & Westbrook J. (in press). Social particiation as an indicator of successful aging: an overview of concepts and their associations with health, *Australian Health Review.*

Draper, BM. (2014). Suicidal behaviour and suicide prevention in later life. *Maturitas*, 79, 179-83.

Golden J, Conroy RM, Bruce I, Denihan A, Greene E, Kirby M, & Lawlor BA. 2009. Loneliness, social support networks, mood and wellbeing in community-dwelling elderly. *International Journal of Geriatric Psychiatry*, 24, 694-700.

Gould RL, Coulson MC, Howard RJ (2012a). Cognitive behavioral therapy for depression in older people: a meta-analysis and meta-regression of randomized controlled trials. *Journal of the American Geriatrics Society* 60, 1817–1830.

Gould RL, Coulson MC, Howard RJ (2012b). Efficacy of cognitive behavioral therapy for anxiety disorders in older people: a meta-analysis and meta-regression of randomized controlled trials. *Journal of the American Geriatrics Society* 60, 218–229.

Gum AM, Arean PA, Bostrom A 2007. Low-income depressed older adults with psychiatric co-morbidity: secondary analyses of response to psychotherapy and case management. *International Journal of Geriatric Psychiatry* 22, 124–130.

Hodgetts S, Gallagher P, Stow D, Ferrier N, & O'Brian JT. (2017). The impact and measurement of social dysfunction in late-life depression: an evaluation of current methods with a focus on wearable technology. *International Journal of Geriatric Psyhiatry*, 32, 247-255.

Hsieh, S., Schubert, S., Hoon, C., Mioshi, E., and Hodges, J.R., (2013). Validation of the Addenbrooke's Cognitive Examination III in frontotemporal dementia and Alzheimer's disease. *Dement Geriatr Cogn Disord*; 36: 242-250.

John, O. P., & Srivastava, S. (1999). The Big-Five trait taxonomy: History, measurement, and theoretical perspectives. In L. A. Pervin & O. P. John (Eds.), Handbook of personality: Theory and research (Vol. 2, pp. 102–138). New York: Guilford Press.

Joiner Jr TE. 2002. Depressive Symptom Index Suicidality Subscale. *Behaviour Research and Therapy*, 40, 471–481.

Krueger RF, Derringer J, Markon KE, Watson D, Skodol AE. (2013). *Measure: The Personality Inventory for DSM-5—Brief Form (PID-5-BF)—Adult*. American Psychiatric Association.

Lenze EJ, Mulsant BH, Shear MK, Schulberg HC, Dew MA...Reynolds CF. 2000. Comorbid anxiety disorders in depressed elderly patients. *American Journal of Psychiatry*, 157:722– 728.

Moher D, Schulz K, & Altman D. (2001). The CONSORT statement: revised recommendations for improving the quality of reports of parallel-group randomised trials. *Lancet, 357,* 1191-94.

Norton S, Matthews FE, Barnes DE, Yaffe K, & Brayne C. (2014). Potential for primary prevention of Alzheimer's disease: an analysis of population-based data. *Lancet Neurology, 13,* 788-94.

Oude Voshaar RC, van der Veen DC, Hunt I, & Kupur N. 2016. Suicide in late-life depression with and without comorbid anxiety disorders. International *Journal of Geriatric Psychiatry*, 31, 146-152.

Paap DPT, Schrier EPT, & Dijkstra PTPU. (2018) Development and validation of the Working Alliance Inventory Dutch version for use in rehabilitation setting, *Physiotherapy*

Theory and Practice, DOI: 10.1080/09593985.2018.1471112

PachanaNA, ByrneGJ, SiddleH, KoloskiN,Harley E,Arnold E (2007).Development and validation of the GeriatricAnxiety Inventory. *International Psychogeriatrics* 19, 103–114.

Perrissinotto CM et al. 2015. Loneliness in older persons: A predictor of functional decline and death. *Archives of Internal Medicine*, 172 (14), 1078-1083. http://refhub.elsevier.com/S0920-9964(17)30328-6/rf0095

Segal DL, June A., Coolidge FL., Yochim B. (2010).Development and initial validation of a self-report assessment tool for anxiety among older adults: the Geriatric Anxiety Scale. *Journal of Anxiety Disorders,* 24(7), 709-714, http://doi: 10.1016/j.janxdis.2010.05.002

van der Gaag M, Schütz C, Ten Napel A, Landa Y, Delespaul P, Bak M, Tschacher W, de Hert M. (2013). Development of the Davos assessment of cognitive biases scale (DACOBS). Schizophrenia Research, 144(1-3), 63-71, doi: 10.1016/j.schres.2012.12.010. Epub 2013 Jan 15.

Waltz J., Addis ME., Koerner K., Jacobson NS. (1993). Testing the integrity of a psychotherapy protocol: Assessment of adherence and competence. *Journal of Consulting and Clinical Psychology,* 61(4), 620-630. http://dx.doi.org/10.1037/0022-006X.61.4.620

Wetherell JL, Thorp SR, Patterson TL, Golshan S, Jeste DV, & Gatz M. (2004). Quality of life in geriatic generalized anxiety disorder: a preliminary investigation. *Journal of Psychiatric Research*, 38, 305-312.

Wuthrich VM, Rapee RM, Kangas M, & Perini S. (2016). Randomised Controlled Trial of Group Cognitive Behavioural Therapy Compared to a Discussion Group for Comorbid Anxiety and Depression in Older Adults. *Psychological Medicine,* 46 (4), 785-795.

Wuthrich VM, & Rapee RM. (2013). Randomized controlled trial of group cognitive behavioral therapy for comorbid anxiety and depression in older adults. *Behaviour Research and Therapy, 51*, 779-786.

Yesavage JA, Brink TL, Rose TL, Lum O, Huang V, Adley M, L eirer VO (1983). Development and validation of a geriatric depression screening scale: a preliminary report. *Journal of Psychiatric Research* 1, 37–49.

18. APPENDICES

List all appendices i.e.:

Appendix 1	Participant Information and Consent Form
Appendix 2.	Overview Questionnaires
Appendix 3.	Advertisements (Client Flyer – GP Flyer)
Appendix 4.	Other
